# Supplementary material for: The influence of environment on bacterial co-abundance in the gut microbiomes of healthy human individuals
Source: Commun Biol. 2025 Nov 6;8:1537. doi: 10.1038/s42003-025-08895-y (PMC12592440; doi:10.1038/s42003-025-08895-y)
Supplement: Supplementary file 3 — Description of Additional Supplementary Materials [file 42003_2025_8895_MOESM3_ESM.pdf]

Description of Additional Supplementary Files

File name: Supplementary Data 1

Description: Environmental and clinical factors from the Milieu Interieur study

File name: Supplementary Data 2

Description: Genus included in the analysis

File name: Supplementary Data 3

Description: Host variables screening

File name: Supplementary Data 4

Description: Univariate association between sex and the top contributing genera
